# Multifaceted role of the Topo IIIα–RMI1-RMI2 complex and DNA2 in the BLM-dependent pathway of DNA break end resection

**DOI:** 10.1093/nar/gku803

**Published:** 2014-09-08

**Authors:** James M. Daley, Tamara Chiba, Xiaoyu Xue, Hengyao Niu, Patrick Sung

**Affiliations:** Department of Molecular Biophysics and Biochemistry, Yale University School of Medicine, New Haven, CT 06520, USA

## Abstract

BLM, a RecQ family DNA helicase mutated in Bloom's Syndrome, participates in homologous recombination at two stages: 5′ DNA end resection and double Holliday junction dissolution. BLM exists in a complex with Topo IIIα, RMI1 and RMI2. Herein, we address the role of Topo IIIα and RMI1-RMI2 in resection using a reconstituted system with purified human proteins. We show that Topo IIIα stimulates DNA unwinding by BLM in a manner that is potentiated by RMI1-RMI2, and that the processivity of resection is reliant on the Topo IIIα–RMI1-RMI2 complex. Topo IIIα localizes to the ends of double-strand breaks, thus implicating it in the recruitment of resection factors. While the single-stranded DNA binding protein RPA plays a major role in imposing the 5′ to 3′ polarity of resection, Topo IIIα also makes a contribution in this regard. Moreover, we show that DNA2 stimulates the helicase activity of BLM. Our results thus uncover a multifaceted role of the Topo IIIα–RMI1-RMI2 ensemble and of DNA2 in the DNA resection reaction.

## INTRODUCTION

DNA double-strand breaks (DSBs) are among the most dangerous DNA lesions, as they can potentially lead to cell death or gross chromosomal rearrangements that characterize cancer cells. DSB repair occurs via two distinct mechanisms, homologous recombination (HR) and nonhomologous end joining (NHEJ). In NHEJ, the DNA break ends are aligned, processed if necessary and religated (1,2). HR is more complex mechanistically, entailing extensive, unidirectional resection of the DNA break ends to yield a pair of 3′ single-strand DNA (ssDNA) tails. One of these tails then invades a homologous sequence to form a displacement loop or D-loop. Subsequent steps include DNA synthesis initiating from the primer template junction in the D-loop and resolution of the extended DNA structure via one of several pathways. In all the known HR pathways, the commencement of DNA end resection signifies cellular commitment to break repair via HR (3,4). Moreover, ssDNA stemming from resection triggers activation of the ATR/ATRIP DNA damage checkpoint (5).

Genetic analyses have unveiled surprising complexity of the DNA end resection process, with three distinct nucleases being involved (6,7). The MRE11 nuclease, acting within the context of the MRE11-RAD50-NBS1 (MRN) complex, initiates resection in concert with its partner CtIP (8). Further resection is mediated via two, non-overlapping pathways, one being catalyzed by the 5′ to 3′ exonuclease EXO1, and the other by the Bloom's syndrome (BLM) helicase with the nuclease DNA2 (9–11). The efficiency of DNA end resection is enhanced by the ssDNA binding protein RPA and MRN through interactions with components of the EXO1 and BLM-DNA2 resection machineries (12–14). Moreover, *in vitro* studies have revealed that RPA–DNA2 interaction helps impose the 5′ polarity of resection (15,16).

BLM associates with Topo IIIα, RMI1 and RMI2 (the Topo IIIα–RMI1-RMI2 complex is called the TR complex) in cells (17–20). The higher order ensemble of BLM-Topo IIIα-RMI1-RMI2 dissolves the double Holliday junction at a late stage of HR to prevent a crossover outcome, and the role of the TR complex herein has been studied recently (21–26). In contrast, whether the TR complex is needed for DNA end resection and, if so, the mechanism by which it influences resection efficiency have remained unknown.

We have developed a reconstituted system to define the function of the TR complex in DNA end resection, and here we provide evidence for a multifaceted role of this complex in the reaction. Moreover, we have unveiled a role of DNA2 in BLM stimulation. Our reconstituted system should be valuable for the continual dissection of the mechanism of DNA end resection in human cells.

## MATERIALS AND METHODS

### Protein purification

Affinity epitope-tagged forms of BLM, Sgs1, Top3, WRN, Topo IIIα and RMI1-RMI2 were expressed and purified as described previously (16,23,24,27). DNA2 with a C-terminal Flag epitope was cloned into the NcoI and XhoI sites in pFastBacHTB (Life Technologies) to create pJD72, and a bacmid was generated in the *Escherichia coli* strain DH10Bac (Invitrogen). The bacmid was used to generate a recombinant baculovirus in SF9 insect cells. HighFive insect cells at a density of 1 × 10^6^ cells/ml were infected with the baculovirus and harvested after 48 h of incubation in shaking flasks. All the subsequent steps were carried out between 0 and 4°C. An extract was prepared by sonication of a cell pellet derived from 600 ml of culture in 100 ml of K buffer (20 mM KH_2_PO_4_, 10% glycerol, 0.5 mM EDTA, 0.01% IGEPAL, 1 mM DTT, 1 mM phenylmethylsulfonyl fluoride and 5 μg/ml each of aprotinin, chymostatin, leupeptin and pepstatin) containing 300 mM KCl. After ultracentrifugation (100 000 × g for 60 min), the clarified lysate was diluted with K buffer to a KCl concentration of 150 mM and then fractionated in a 10 ml SP Sepharose column (Amersham) using a 100 ml gradient of 150–650 mM KCl. DNA2-containing fractions were pooled and incubated with 2 ml of anti-Flag M2 agarose (Sigma) for 1 h with constant agitation. The resin was washed with 15 ml of K buffer with 300 mM KCl, 15 ml of K buffer with 1M KCl, 1 mM ATP and 8 mM MgCl_2_ and 15 ml of K buffer with 150 mM KCl. To elute the bound proteins, the resin was treated for 30 min with 1 ml of K buffer containing 150 mM KCl and 250 ng/μl of Flag peptide (Sigma). This elution procedure was repeated four additional times. The pooled eluate was loaded onto a 1 ml Source Q column and eluted with a 50 ml gradient of 150–500 mM KCl. Fractions containing DNA2 were pooled, diluted to 150 mM KCl with K buffer and fractionated in a Heparin column (Amersham) with a 50 ml gradient of 150–1000 mM KCl. Fractions containing the DNA2 peak were combined and concentrated to 2 μM using Amicon filter tubes and stored in small portions at −80°C. The DNA2-D277A and DNA2-D277A/K654E mutants were generated by QuickChange (28,29) and purified using the same protocol.

EXO1 with a C-terminal Flag epitope was introduced into the ClaI site in pESC-Ura (Agilent) to create pJD124 in the *Saccharomyces cerevisiae* strain YRP654 (30). Yeast cells were grown at 30°C for 12 h in the presence of 2% galactose to induce EXO1 expression. All the subsequent steps were carried out between 0 and 4°C. Cells were disrupted using a Krups coffee grinder and resuspended in 150 ml of K buffer (20 mM KH_2_PO_4_, 10% glycerol, 0.5 mM EDTA, 0.01% IGEPAL, 1 mM DTT, 1 mM phenylmethylsulfonyl fluoride and 5 μg/ml each of aprotinin, chymostatin, leupeptin and pepstatin) containing 300 mM KCl. After ultracentrifugation (100 000 × g for 60 min), the clarified lysate was diluted with K buffer to a KCl concentration of 150 mM and then loaded onto a 10 ml Q Sepharose column (Amersham), washed with 30 ml of K buffer with 150 mM KCl and eluted into K buffer containing 500 mM KCl. The elution was diluted with an equal volume of K buffer and incubated with 1 ml of anti-Flag M2 agarose (Sigma) for 90 min with constant agitation. The matrix was washed with 15 ml of K buffer with 250 mM KCl, 15 ml of K buffer with 500 mM KCl, 1 mM ATP and 8 mM MgCl_2_ and 15 ml of K buffer with 150 mM KCl. To elute bound proteins, the matrix was treated for 30 min with 1 ml of K buffer containing 150 mM KCl and 250 ng/μl of Flag peptide (Sigma). This elution procedure was repeated four additional times. The pooled eluate was loaded onto a 4 ml Source S column and eluted with a 60 ml gradient of 150–650 mM KCl. Fractions containing EXO1 were pooled, diluted to 150 mM KCl with K buffer and fractionated in a Heparin column (Amersham) with a 50 ml gradient of 150–650 mM KCl. Fractions containing the EXO1 peak were combined and concentrated to 1.6 μM using Amicon filter tubes and stored in small portions at −80°C.

The identity of DNA2 and EXO1 was established by immunoblot analysis using anti-Flag antibodies (Sigma), and its elution from different matrices was monitored by 7.5% SDS-PAGE and Coomassie Blue staining.

### DNA substrates

The oligonucleotides used to make DNA substrates are listed in Supplementary Table S1. The 2 kb dsDNA substrate is a fragment of the DNA2 gene created by PCR with oligonucleotides PSOL4642 and PSOL6134 using pJD72 as template; 50 μCi of α^32^P-dCTP (Perkin-Elmer) was included in the reaction to randomly radiolabel the PCR fragment. The 80-base dsDNA substrate used in Figure 2A was created by annealing complementary oligonucleotides PSOL1239 and PSOL2243, and 5′ biotinylated versions of these oligonucleotides, PSOL7475 and PSOL6214, respectively, were used to create the substrates in Figure 2B, and were incubated with 0.5 mg/ml streptavidin for 10 min at 25°C. The 315 bp dsDNA substrate used in the atomic force microscopy (AFM) experiments in Figure 2C and D was created by PCR with oligonucleotides PSOL7112 and PSOL7113 on the template pJD87, which was made by cloning the human SSBIP1 coding sequence into the BglII and KpnI sites of pET-Duet. The Y DNA substrates in Figure 4C–E were created by annealing oligonucleotides PSOL7287 and PSOL7288, or PSOL7345 and PSOL7346, to give the substrates that harbor the 19 or 44 base overhangs, respectively. DNA substrates that harbor either a 3′ or 5′ overhang in Figure 4A and B were created by annealing oligonucleotides PSOL7287 and PSOL7369 (19-base 5′ overhang), PSOL7379 and PSOL7288 (19-base 3′ overhang), PSOL7343 and PSOL7369 (44-base 5′ overhang) and PSOL7370 and PSOL7344 (44-base 3′ overhang). The indicated oligonucleotide in each substrate was 5′ end labeled with γ^32^P -ATP (Perkin-Elmer) and T4 polynucleotide kinase (NEB) or 3′ end labeled with α^32^P-dATP (Perkin-Elmer) and terminal transferase (NEB) before annealing. All the substrates were purified by electrophoresis in native 10% polyacrylamide gels run in TBE buffer (90 mM Tris, 90 mM boric acid, 0.5 mM EDTA, pH 8.0) at 4°C and electroelution into the same buffer.

**Figure 1.**
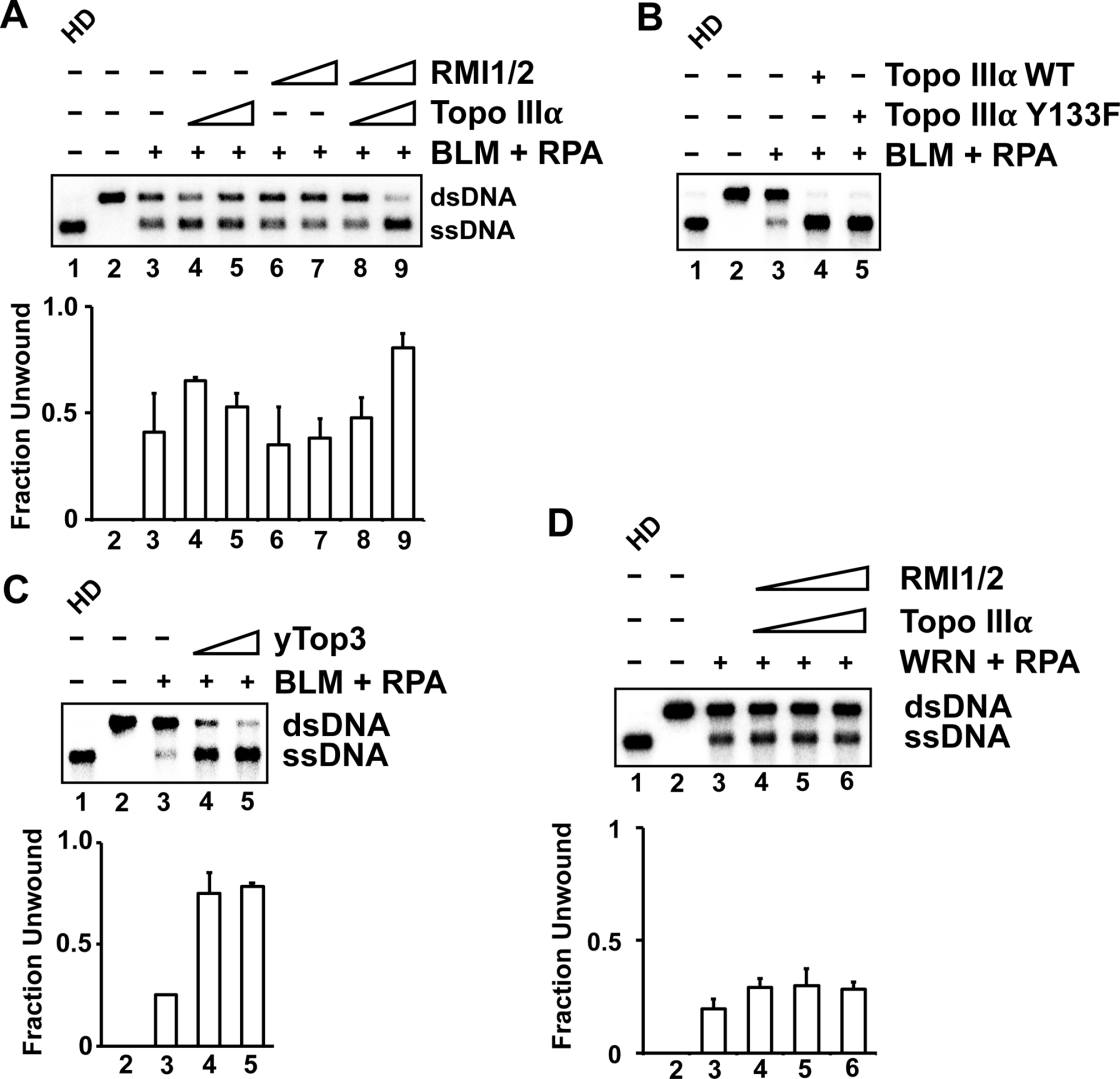
Topo IIIα-RMI1-RMI2 stimulates DNA unwinding by BLM-RPA. (**A**) The effects of Topo IIIα (1 or 5 nM) and RMI1-RMI2 (1 or 5 nM) on the unwinding of a 2 kb randomly radiolabeled dsDNA (0.5 nM ends) by BLM (1 nM) and RPA (200 nM) were examined. The reaction was incubated at 37°C for 30 min. In lane 1, the DNA was heat denatured (HD) by boiling for 2 min. The data shown are the average from three independent experiments with the error bars representing one standard deviation. (**B**) Topo IIIα (wild-type or Y133F) (5 nM) was incubated with BLM (2.5 nM) and RPA (200 nM) for 30 min at 37°C. (**C**) Yeast Top3 (1 or 5 nM) was incubated with BLM (4 nM) for 10 min at 37°C. (**D**) TR (15, 30 or 60 nM) was incubated with WRN (30 nM) and RPA (200 nM) for 30 min at 37°C.

**Figure 2.**
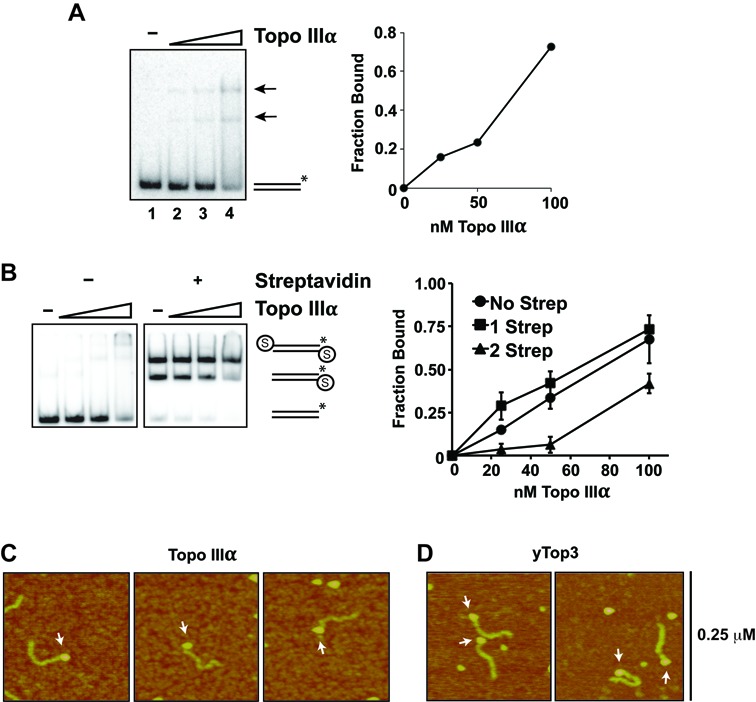
Recognition of DNA ends by Topo IIIα. (**A**) Topo IIIα (25, 50 or 100 nM) was incubated with radiolabeled 80-mer dsDNA (5 nM ends). Quantification of DNA binding is shown in the right panel. The arrow denotes nucleoprotein complexes and the asterisk indicated the location of the radiolabel in the DNA substrate. (**B**) DNA mobility shift was conducted as in (A), except that dsDNA substrate blocked by biotin-streptavidin (denoted by the circled S) at either one or both of the ends was used. The data shown were the average from three independent experiments and the error bars represent 1 SD. (**C**, **D**) AFM was used to image nucleoprotein complexes of Topo IIIα and Top3. Arrows indicate DNA end binding events.

**Figure 3.**
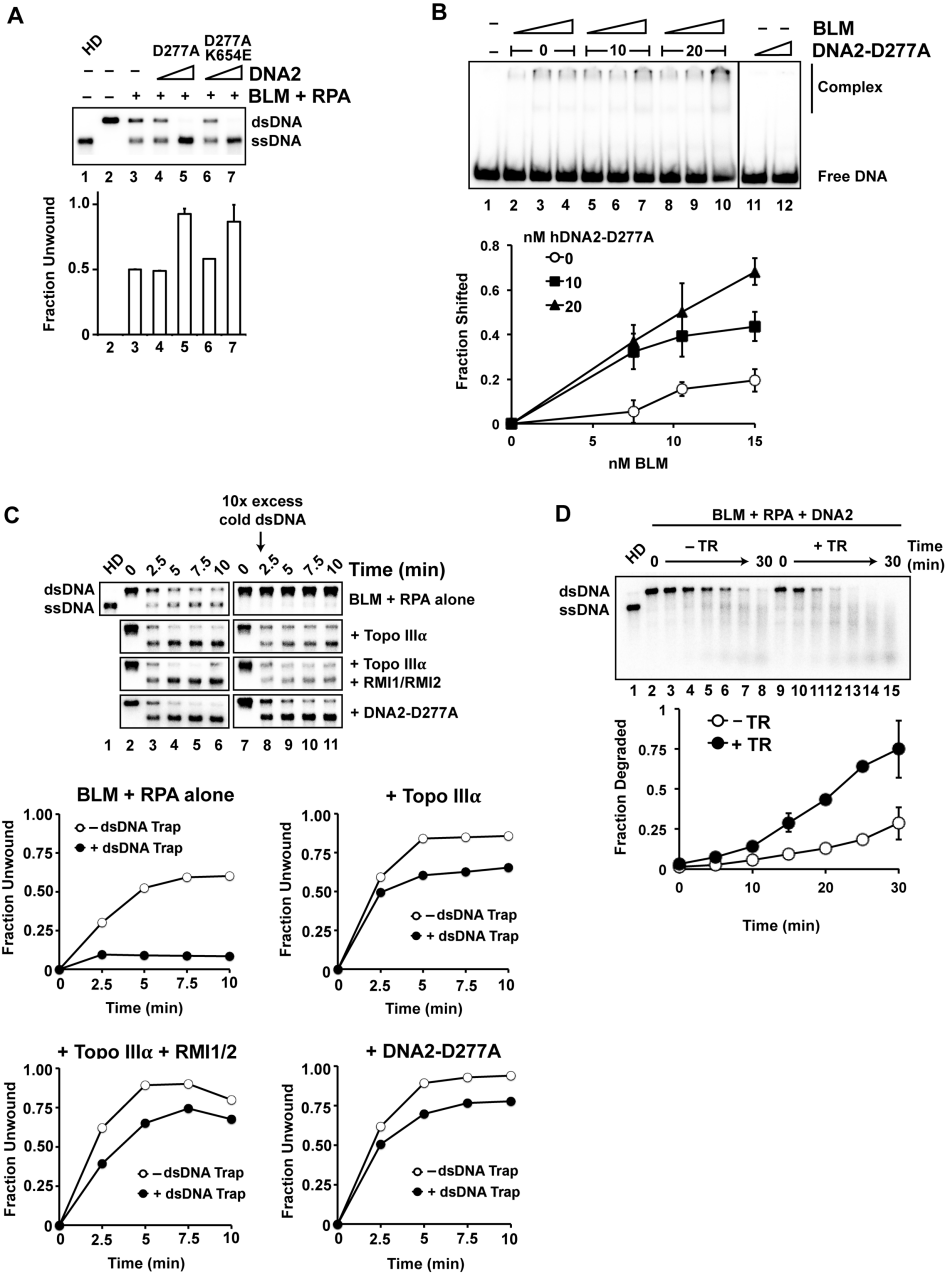
Stimulation of BLM helicase processivity by the TR complex and DNA2-D277A. (**A**) BLM-mediated DNA unwinding was examined with 5 nM BLM and the addition of DNA2-D277A or DNA2-D277A/K654E (5 or 20 nM). Reactions were incubated for 10 min at 37°C (**B**) Binding of an 80-mer dsDNA by BLM (7.5, 10.5 or 15 nM) without or with DNA2-D277A (10 or 20 nM). The data shown are the average from three independent experiments and the error bars represent 1 SD. (**C**) A randomly radiolabeled 2 kb dsDNA (0.5 nM ends) was incubated with BLM (5.5 nM) and RPA (200 nM), and 10-fold excess unlabeled dsDNA (identical to the labeled substrate in sequence) was added at the 2-min point along with the TR complex (5 nM) or DNA2-D277A (16.5 nM). Samples were taken at 2.5 min intervals and analyzed. Quantification of the results is shown below. (**D**) Resection of a 2 kb dsDNA (0.5 nM ends) by BLM (2 nM), DNA2 (10 nM) and RPA (200 nM) was examined without or with the TR complex (5 nM). The average data from three independent experiments are shown and the error bars represent 1 SD.

**Figure 4.**
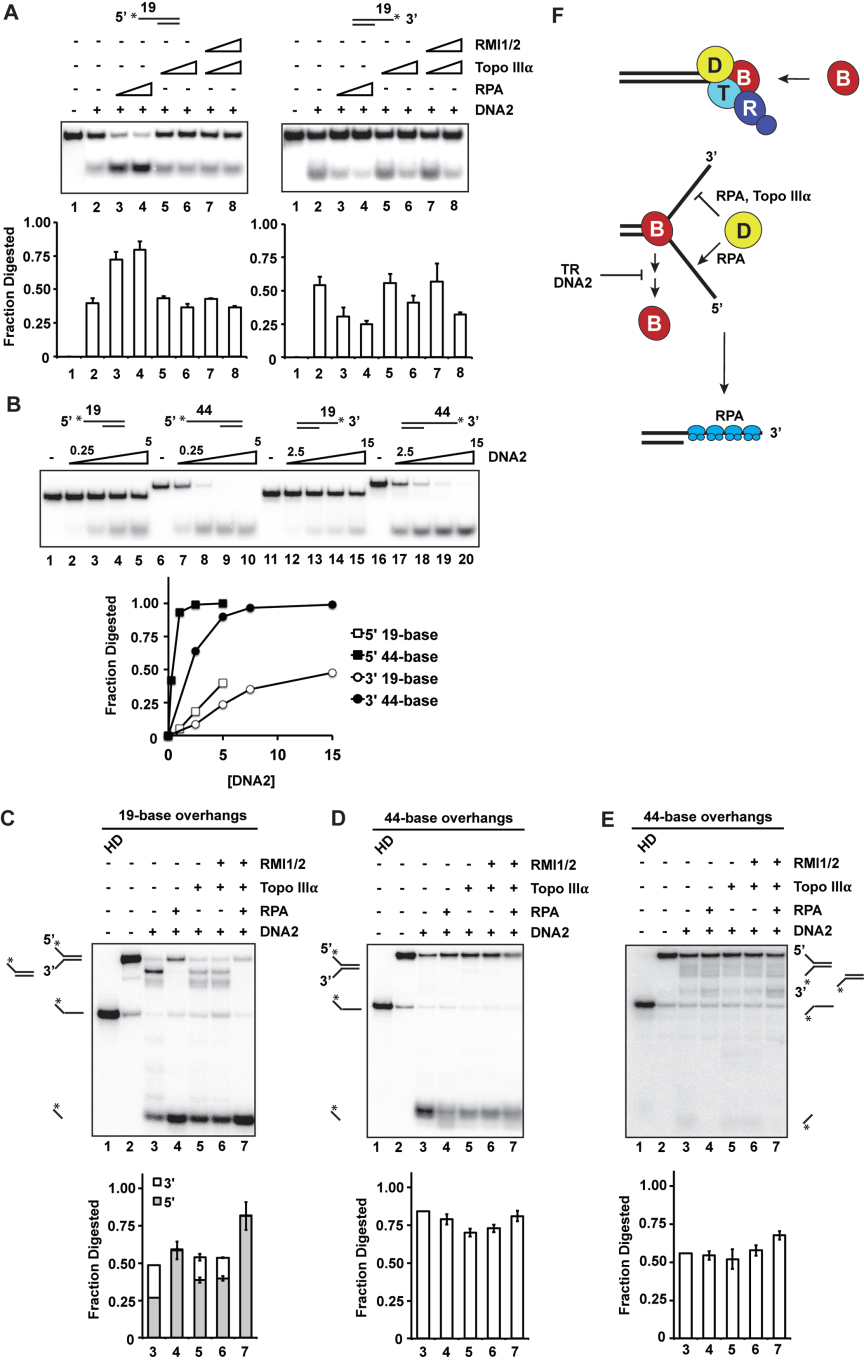
Effect of Topo IIIα and RMI1-RMI2 on DNA2 endonuclease activity. (**A**) DNA2 (10 or 20 nM protein for the substrates with the 5′ or 3′ overhang, respectively) was incubated with RPA (5 or 10 nM) or Topo IIIα (1 or 5 nM) and RMI1-RMI2 (1 or 5 nM) for 15 min at 30°C. The average data from three independent experiments are shown and the error bars represent 1 SD. The radiolabel is denoted by the asterisk. (**B**) DNA substrates with either a 19- or 44-nt 3′ or 5 overhang were incubated with DNA2 at the indicated concentrations. The radiolabel is denoted by the asterisk. (**C**) DNA2 (5 nM), RPA (5 nM), Topo IIIα (30 nM) and RMI1-RMI2 (30 nM) were incubated with Y DNA substrates containing 19-base overhangs for 20 min at 30°C. The radiolabel is denoted by the asterisk. Degradation products are pictured adjacent to their positions on the gel. (**D**, B) DNA2 (1 nM), RPA (5 nM), Topo IIIα (30 nM) and RMI1-RMI2 (30 nM) were incubated with a forked DNA substrate containing 44-base overhangs. The substrate was labeled (denoted by the asterisk) on the end of the 5′ overhang (D) or 3′ overhang (**E**). (**F**) Model for the multifaceted role of the TR complex and DNA2 in end resection. Topo IIIα and DNA2 contribute to BLM recruitment to DNA ends (top), and when unwinding begins, TR and DNA2 both enhance the processivity of BLM (bottom), where the double arrow indicates dissociation of BLM from the DNA. RPA enforces the 5′ to 3′ directionality of resection by modulating the endonuclease activity of DNA2 (bottom), with Topo IIIα also contributing in this regard.

### Helicase and nuclease assays

Helicase and nuclease assays were performed in buffer R (20 mM Na-HEPES pH 7.5, 2 mM ATP, 0.1 mM DTT, 100 μg/ml BSA, 0.05% Triton-X 100, 2 mM MgCl_2_, 100 mM KCl) and contained 0.5 nM ends (2 kb substrate) or 5 nM ends (all other substrates) of DNA. Reactions containing BLM or WRN also included an ATP regenerating system consisting of 10 mM creatine phosphate and 50 μg/ml creatine kinase. The reactions were incubated at 37°C for 10 min, except when assaying for the DNA2 nuclease activity (Figure 4), in which case the reactions were incubated at 30°C for 15 min. After the addition of SDS to 0.02%, proteinase K to 0.25 μg/μl and 0.08% Orange G dye with a final glycerol concentration of 10%, the reaction mixtures were incubated for 5 min at 37°C. Electrophoresis was done in a native 10% polyacrylamide gel in TBE buffer or in a 1% agarose gel in TAE buffer (40 mM Tris-acetate, pH 7.4, 0.5 mM EDTA). Gels were dried onto DEAE paper on top of Whatman filter paper (GE) and then analyzed in a BioRad Personal Molecular Imager FX phosphorimager.

### DNA mobility shift assay

Proteins were incubated with the indicated DNA substrate (5 nM DNA ends) in DNA binding buffer (35 mM Tris-HCl, pH 7.5, 1 mM DTT, 100 ng/μl BSA, 2 mM MgCl_2_, 100 mM NaCl) at 25°C for 20 min, followed by analysis in 6% polyacrylamide gels in TBE buffer and phosphorimaging analysis, as above.

### Atomic force microscopy

Freshly cleaved mica was functionalized with 1-(3-Aminopropyl)silatrane (APS) according to a published method (31–33), resulting in a positively charged surface for DNA retention. Briefly, 4 × 1 cm cleaved mica sheets were placed into plastic cuvettes. Stock APS solution (50 mM) was diluted 1:300 in water and poured into the cuvettes. After 30 min, the mica was rinsed thoroughly with water and dried with a stream of argon and then placed under vacuum for 5 h before use. The DNA substrate was a 315 bp PCR product (described above). DNA (5 nM ends) was incubated with 25 nM human Topo IIIα or yeast Top3 in buffer R lacking BSA at 25°C for 15 min. The reaction was diluted with four volumes of buffer R, 5 μl of which was immediately deposited onto precut APS-mica (1 × 1 cm). After 2 min, the mica was rinsed with water, dried with argon and placed in a vacuum overnight. Images were acquired in tapping mode in air using a Veeco Nanoscope IIIa controller (Veeco) and silicon-etched cantilevers (Asylum Research) with a nominal spring constant of 42 N/m and resonant frequency of 300 kHz. Images were analyzed either with Veeco offline software or Femtoscan Online. For quantification, the total number of DNA molecules counted was 1003 for Topo IIIα and 146 for yeast Top3.

## RESULTS

### Enhancement of BLM-mediated DNA unwinding by Topo IIIα and the TR complex

In the BLM-DNA2 resection pathway, BLM unwinds dsDNA, and the 5′ ssDNA strand in the unwound structure is cleaved by DNA2 (12). We expressed Topo IIIα and RMI1-RMI2 in *E. coli* and purified them to near homogeneity (18,23) to ask whether they could stimulate dsDNA unwinding by BLM. We observed stimulation of BLM-mediated DNA unwinding by Topo IIIα but not RMI1-RMI2 (Figure 1A). However, the TR complex proved to be more effective than Topo IIIα alone (Figure 1A). The topoisomerase defective Topo IIIα Y133F mutant was just as effective as wild-type in this regard (Figure 1B). We note that yeast Top3, the Topo IIIα ortholog, is able to stimulate human BLM, indicating functional conservation (Figure 1C). Importantly though, TR has no effect on the related RecQ family helicase WRN (Figure 1D).

### DNA end recognition by Topo IIIα

We next explored the idea that TR helps target BLM to DNA ends. For this, DNA electrophoretic mobility shift was carried out to examine the interaction of Topo IIIα and RMI1-RMI2 with an 80 bp dsDNA substrate. Topo IIIα formed two discrete nucleoprotein complexes with the substrate (Figure 2A), whereas no substrate shift occurred with RMI1-RMI2 alone (Supplementary Figure S1A) (24,34).

One interpretation of the Topo IIIα gel shift pattern (Figure 2A) is that it associates with the DNA ends. To test this premise, Topo IIIα was incubated with a mixture of dsDNA with one or both of the ends blocked by a biotin–streptavidin complex. Topo IIIα shifted the substrate with only one blocked end efficiently (Figure 2B), but when both ends were blocked, the substrate became shifted only at the highest concentration of Topo IIIα (20-fold excess over DNA) (Figure 2B). Thus, Topo IIIα appears to specifically recognize DNA ends. Finally, we applied AFM to image both human Topo IIIα and yeast Top3 bound to linear dsDNA to determine whether there is specific engagement of DNA ends. Importantly, 70% of the nucleoprotein complexes formed by Topo IIIα had the protein associated with a DNA end (Figure 2C), with the remaining 30% showing internal binding (Supplementary Figure S1B). With Top3, 89% of the nucleoprotein complexes examined had the protein located at a DNA end (Figure 2D) and 11% were bound internally. Collectively, these results show that Topo IIIα and Top3 possess a specific affinity for DNA ends.

### Stimulation of BLM helicase by DNA2

We expressed wild-type DNA2, the nuclease-deficient D277A mutant and the D277A/K654E mutant that is also impaired for helicase activity, in insect cells and purified them to near homogeneity (Supplementary Figure S2A). We saw evidence of increased ssDNA intermediates when DNA2 was included in a reaction with BLM, suggesting that DNA2 either contributes to DNA unwinding or enhances the helicase activity of BLM. The effect was difficult to quantify, however, because of digestion of the unwound DNA by DNA2. Importantly, enhanced DNA unwinding also occurred when BLM was incubated with the D277A or D277A/K654E mutant (Figure 3A), while DNA2-D277A alone did not unwind the dsDNA (Supplementary Figure S2B). These results indicate that DNA2 enhances the DNA helicase activity of BLM. This effect of DNA2 is specific, as the related 5′ flap endonuclease FEN-1 was unable to stimulate BLM (Supplementary Figure S2C). We note that enhancement of BLM-mediated DNA unwinding by DNA2-D277A requires the presence of RPA (Supplementary Figure S2D).

DNA2 on its own does not have sufficient affinity for dsDNA to produce a gel mobility shift (Figure 3B, lanes 11 and 12), but we considered the possibility that the DNA2–BLM complex may have a higher affinity for dsDNA than BLM alone. Consistent with this idea, when tested under conditions wherein ATP and magnesium were omitted to prevent DNA unwinding by BLM, DNA2-D277A enhanced binding of the dsDNA by BLM (Figure 3B). We note that DNA2-D277A retains the ability to bind DNA substrates that harbor either a 3′ or 5′ overhang (Supplementary Figure S2E). Thus, like the TR complex, DNA2 stimulates the helicase activity of BLM, likely by increasing the affinity of BLM for DNA.

### Enhancement of BLM helicase processivity by the TR complex and DNA2

Next, we asked whether TR and DNA2 enhance the processivity of the BLM helicase activity. For this, we adapted the experimental approach previously used to assess the processivity of EXO1 in the DNA mismatch repair reaction (35). Specifically, BLM and RPA were incubated with a 2 kb radiolabeled linear dsDNA substrate, and DNA unwinding was monitored over a 10-min time course (Figure 3C). Experiments were performed with or without the addition of a 10-fold excess of unlabeled dsDNA 2 min into the reaction to trap BLM that had dissociated from the labeled DNA. Indeed, unwinding of the labeled dsDNA by BLM was strongly inhibited upon addition of the DNA trap (Figure 3C, top panel). This result is consistent with previous reports that BLM is relatively non-processive as a helicase (36,37), and implies that complete separation of the strands in the 2 kb substrate likely entails the dissociation of BLM from and its re-association with the partially unwound substrate.

Importantly, in addition to their stimulatory effect on BLM-mediated DNA unwinding without the DNA trap, Topo IIIα, the TR complex and DNA2-D277A also helped alleviate the inhibitory effect of the competitor DNA (Figure 3C, right panels). Since Topo IIIα, TR and DNA2-D277A were added concurrently with the DNA trap in these experiments, their effect on BLM likely reflects the stabilization of BLM on the partially unwound radiolabeled DNA. We note that neither TR nor DNA2-D277A could stimulate DNA unwinding when the DNA trap was added at the beginning of the reaction (Supplementary Figure S3A).

### Enhancement of DNA resection by the TR complex

We next sought to assess the functional relevance of the TR complex in the fully reconstituted DNA end resection reaction. The TR complex was added together with BLM, RPA and DNA2 and degradation of randomly radiolabeled dsDNA was monitored over a 30-min time course. Importantly, DNA end resection occurred at a greatly elevated rate in the presence of the TR complex (Figure 3D). In contrast, TR did not stimulate resection by EXO1 (Supplementary Figure S3B), indicating that its effect is specific to the BLM-DNA2 resection pathway.

### Attenuation of the 3′ endonuclease activity of DNA2 by Topo IIIα

RPA helps impose the 5′ to 3′ directionality of resection by stimulating the 5′ while attenuating the 3′ endonuclease activity of DNA2 (12,16). We investigated whether the TR complex might also affect the nuclease activity of DNA2. On substrates with a 19-nucleotide overhang, RPA enhanced the 5′ endonuclease activity of DNA2 while inhibiting 3′ cleavage (Figure 4A). Interestingly, Topo IIIα had no effect on 5′ endonuclease activity, but inhibited 3′ DNA cleavage (Figure 4A). Addition of RMI1-RMI2 did not affect the selectivity or efficiency of DNA cleavage (Figure 4A). We considered the possibility that protection of the 3′ DNA overhang by Topo IIIα might stem from preferential binding of this substrate. However, DNA binding analysis revealed the same affinity of Topo IIIα for substrates with either a 3′ or 5′ overhang (Supplementary Figure S4).

The extent to which the DNA strands are separated before 5′ cleavage occurs is unknown, so we extended the ssDNA overhang to 44 nucleotides and asked whether this would affect DNA2 activity. Increasing the overhang length led to higher DNA2 activity on both the 3′ and 5′ overhangs (Figure 4B). Consistent with previous results (28), DNA2 preferentially incised the 5′ DNA strand, especially when the DNA length was increased from 19 to 44 nucleotides (Figure 4B).

To further explore the effect of TR and RPA on DNA2 activity, we created Y-shaped structures resembling partially unwound dsDNA (Figure 4C). We first tested cleavage of the substrate with 19-base overhangs. With DNA2 alone, 5′ and 3′ cleavage products were observed (Figure 4C, lane 3). In agreement with previous results (12), RPA strongly inhibited 3′ cleavage and enhanced 5′ cleavage (Figure 4C, lane 4). Consistent with our earlier observations (Figure 4A), Topo IIIα attenuated 3′ DNA cleavage, and addition of RMI1-RMI2 had little or no effect (Figure 4C, lanes 5–6). We next asked how DNA2 would act on a Y structure with 44-base overhang. Indeed, 5′ cleavage was more efficient with this structure, requiring about 5-fold less DNA2 to achieve the same level of cleavage as on the substrate with the shorter overhangs (Figure 4D, lane 3). Interestingly, RPA did not significantly elevate the efficiency of 5′ cleavage with this substrate (Figure 4D, lane 4). Moreover, there was little digestion of the 3′ overhang by DNA2 (Figure 4E, lane 3), and the addition of RPA and TR further attenuated the small amount of cleavage that we detected (Figure 4E, lanes 4–7).

## DISCUSSION

In cells, DSB end resection is initiated by the MRE11 nuclease, part of the MRN complex, together with CtIP (7,38). After ssDNA is generated near the break site, the BLM-DNA2-dependent pathway functions in parallel with the 5′ to 3′ exonuclease EXO1 to catalyze long-range resection, generating a long stretch of RPA-coated ssDNA, which becomes the substrate for the HR machinery.

Our study has identified novel properties of Topo IIIα/Top3, the TR complex and DNA2 that help elucidate their roles in resection. The principal findings and implications are: (i) Topo IIIα binds DNA ends and therefore likely helps target BLM to DNA, (ii) the DNA end binding activity is conserved in yeast Top3, (iii) the TR complex stimulates the helicase activity of BLM by enhancing its processivity, (iv) DNA2 helps recruit BLM to DNA and similarly stimulates the processivity of BLM, (v) Topo IIIα attenuates the 3′ endonuclease activity of DNA2 to help impose the 5′ to 3′ resection directionality, (vi) enhancement of BLM-mediated DNA unwinding by TR and DNA2 favors 5′ strand cleavage and (vii) like its yeast counterpart Top3 (16), the topoisomerase activity of Topo IIIα is dispensable for its DNA resection roles. We note that the yeast TR (Top3-Rmi1) complex also stimulates Sgs1 helicase activity and resection by Sgs1-Dna2 (15,16), and our results on the human orthologs help us understand the mechanistic basis for the functional synergy (this study).

BLM and Topo IIIα have been shown to interact directly (39), and our study provides mechanistic evidence that this interaction is germane for the recruitment of BLM to DNA ends. The MRE11-RAD50-NBS1 (MRN) complex in humans and its ortholog Mre11-Rad50-Xrs2 (MRX) in *S. cerevisiae* also possess a DNA end binding attribute that is likely relevant for resection (40,41). MRX catalyzes the initial resection that occurs within the vicinity of the DSB and also has been shown to stimulate the long-range resection pathways (12,16). Our work now identifies Topo IIIα as another DNA end targeting element within the BLM-DNA2 reaction pathway. Importantly, whereas MRN stimulates both BLM and EXO1 (12), Topo IIIα appears to be specific for the BLM-DNA2 pathway (this study).

We note that while Topo IIIα alone can enhance the processivity of BLM (Figure 3C and D), the TR complex is more adept in this regard (Figure 1A). Thus, in addition to helping recruit BLM to DNA ends to initiate strand separation, Topo IIIα and the TR complex can also stabilize the association of BLM with a partially unwound substrate. MRN has been shown to similarly enhance the processivity of EXO1 in the resection reaction (12). Therefore, each long-range resection pathway includes a distinct processivity factor.

Besides its influence on DNA unwinding, we have identified a novel role for Topo IIIα in the imposition of 5′ resection polarity. On its own, DNA2 can cleave both 5′ and 3′ DNA overhangs (Figure 4B). RPA was previously shown to help enforce the 5′ to 3′ directionality of resection (12,16). Here, we have presented evidence that Topo IIIα also contributes in this regard. Since Topo IIIα binds substrates with a 3′ or a 5′ overhang equally well (Supplementary Figure S4), it is likely that Topo IIIα exerts its influence by directly modulating the activity of DNA2. We note that enhancement of BLM-mediated unwinding by Topo IIIα-RMI1-RMI2 and DNA2 would lead to an extended Y DNA structure whose 5′ terminus is inherently more amenable to cleavage by DNA2 (Figure 4D and E).

Furthermore, we have shown that DNA2, like TR, stimulates DNA unwinding by BLM (Figure 3A). This attribute of DNA2 is revealed clearly with the use of mutants that are defective in nuclease and helicase activities. Importantly, DNA2 appears to enhance DNA binding by BLM and also the processivity of the BLM helicase function once unwinding has commenced (Figure 3B–D).

Based on our findings, we present a model illustrating the roles of Topo IIIα, RMI1-RMI2 and DNA2 in DNA end resection (Figure 4F). Topo IIIα localizes to DNA ends and helps recruit BLM, with DNA2 also contributing to recruitment. Once DNA unwinding begins, TR and DNA2 enhance the DNA association and hence the processivity of BLM. Finally, the endonuclease activity of DNA2, being guided to the 5′ overhang by RPA and Topo IIIα, cleaves the DNA, resulting in the formation of 3′ ssDNA tails.

## SUPPLEMENTARY DATA

Supplementary Data are available at NAR Online.

SUPPLEMENTARY DATA
