# Supplementary material for: Multifaceted role of the Topo IIIα–RMI1-RMI2 complex and DNA2 in the BLM-dependent pathway of DNA break end resection
Source: Nucleic Acids Res. 2014 Sep 8;42(17):11083–91. doi: 10.1093/nar/gku803 (PMC4176181; doi:10.1093/nar/gku803)
Supplement: SUPPLEMENTARY DATA [file supp_42_17_11083__index.html]

Multifaceted role of the Topo IIIα–RMI1-RMI2 complex and DNA2 in the BLM-dependent pathway of DNA break end resection — Multifaceted role of the Topo IIIα–RMI1-RMI2 complex and DNA2 in the BLM-dependent pathway of DNA break end resection — SUPPLEMENTARY DATA 

# Multifaceted role of the Topo IIIα–RMI1-RMI2 complex and DNA2 in the BLM-dependent pathway of DNA break end resection

## SUPPLEMENTARY DATA

**Files in this Data Supplement:**

- SUPPLEMENTARY DATA
- SUPPLEMENTARY DATA
