# Supplementary material for: Multifaceted role of the Topo IIIα–RMI1-RMI2 complex and DNA2 in the BLM-dependent pathway of DNA break end resection
Source: Nucleic Acids Res. 2014 Sep 8;42(17):11083–91. doi: 10.1093/nar/gku803 (PMC4176181; doi:10.1093/nar/gku803)
Supplement: SUPPLEMENTARY DATA [file supp_gku803_nar-01932-d-2014-File007.pdf]

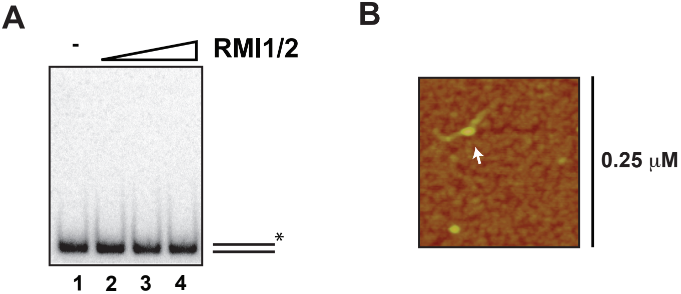

Figure S1. A. RMI1/2 (25, 50, or 100 nM) was incubated with radiolabeled 80-mer dsDNA (5 nM ends). The asterisk indicated the location of the radiolabel in the DNA substrate. B. AFM imaging of Topo IIIα was done as in Figure 2C. A representative image of Topo IIIα binding to dsDNA internally is shown.

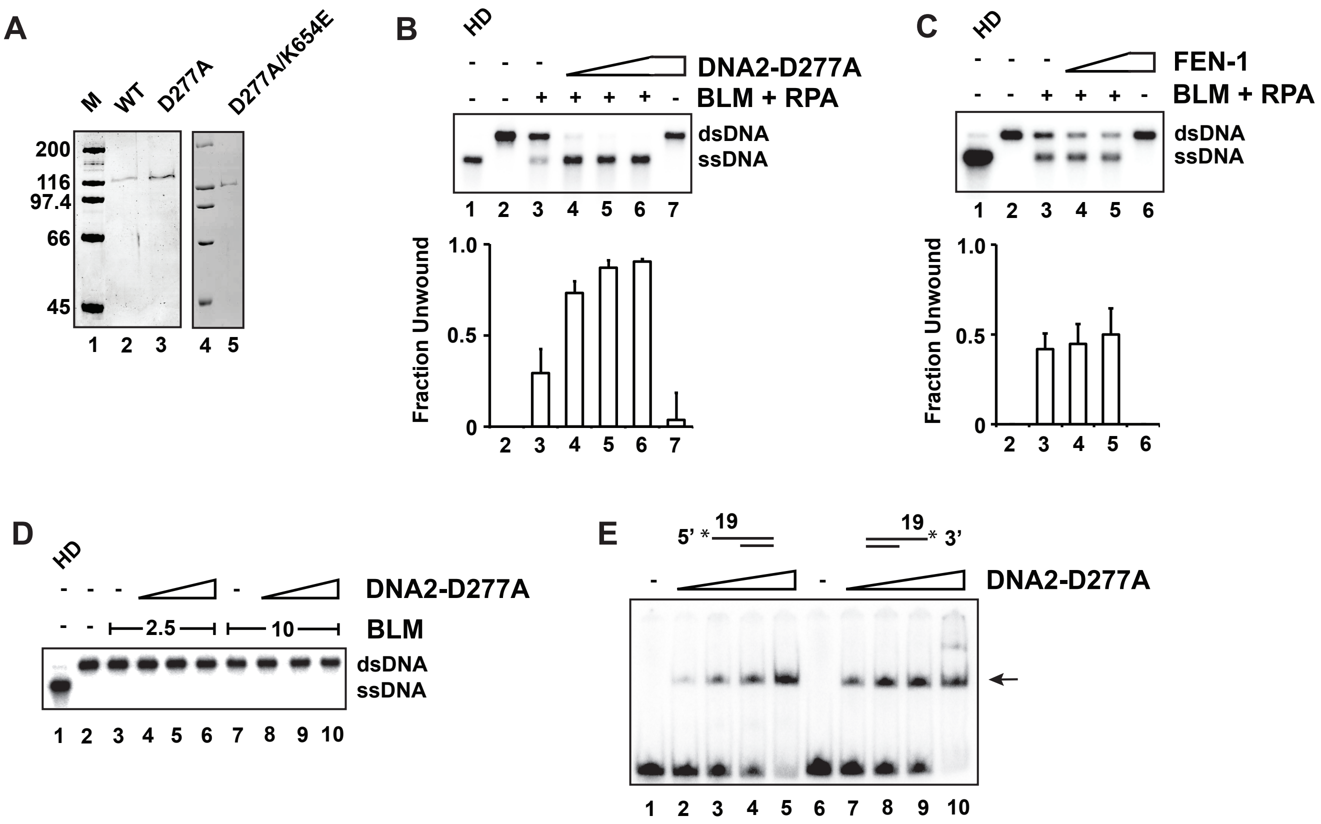

Figure S2. A. DNA2 and the indicated mutants were purified from HighFive insect cells and analyzed on an 8% SDS-PAGE gel stained with Coomassie Blue. B. DNA unwinding was examined as in Figure 1A with 1 nM BLM and the addition of DNA2-D277A (2.5, 5 or 10 nM). C. Same as Figure 3A, except that human FEN1 (1 or 5 nM) was examined with 1 nM BLM. D. RPA is required for stimulation of BLM by DNA2-D277A. DNA2-D277A (0.5, 5 or 20 nM) and BLM (2.5 or 10 nM) were incubated with the 2kb dsDNA substrate (0.5 nM ends) for 30 min at 37°C. E. DNA2-D277A (2.5, 5, 10, or 20 nM) was incubated with the indicated DNA substrate (5 nM ends) for 5 min at room temperature. The asterisk denotes the position of the radiolabel in the substrates, and the nucleoprotein complex is indicated by an arrow.

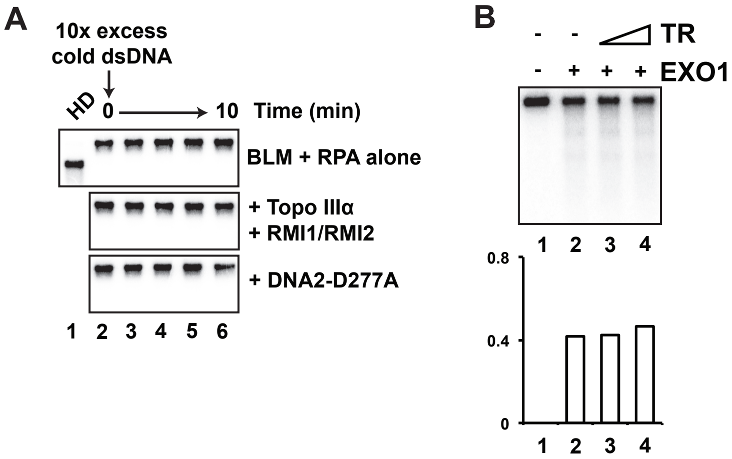

Figure S3. A. Resection reactions were performed as in Figure 3C, except that trap DNA was added at the very beginning of the reaction. B. TR does not stimulate resection by EXO1. Topo IIIα and RMI1-RMI2 (1 or 5 nM) were incubated with EXO1 (5 nM) and randomly radiolabeled 2 kb dsDNA (0.5 nM ends). Quantitation is shown below.

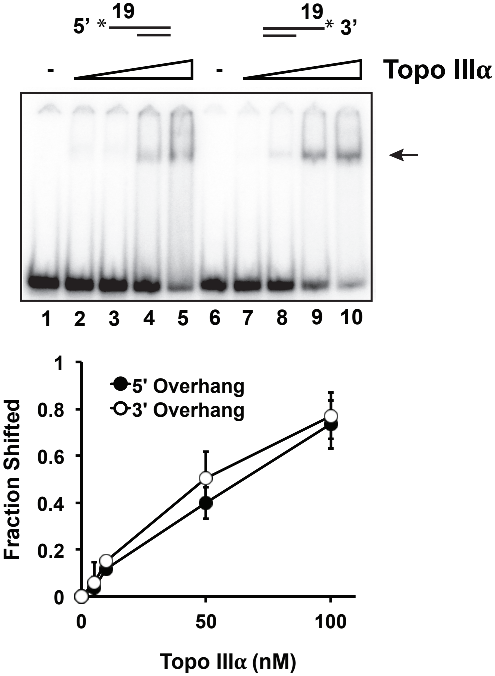

Figure S4. Topo IIIα binds DNA substrates with either a 5' or 3' overhang equally well. Topo IIIα (5, 10, 50 or 100 nM) was incubated with the indicated DNA substrate (5 nM ends). The asterisk denotes the radiolabel in the substrates, and the arrow indicates the nucleoprotein complexes.
