# Supplementary material for: Multifaceted role of the Topo IIIα–RMI1-RMI2 complex and DNA2 in the BLM-dependent pathway of DNA break end resection
Source: Nucleic Acids Res. 2014 Sep 8;42(17):11083–91. doi: 10.1093/nar/gku803 (PMC4176181; doi:10.1093/nar/gku803)
Supplement: SUPPLEMENTARY DATA [file supp_gku803_nar-01932-d-2014-File006.docx]

**Supplemental Table 1:** Sequences of oligonucleotides used in this study.

| Name | Sequence | Description |
| --- | --- | --- |
| PSOL1239 | TTGATAAGAGGTCATTTGAATTCATGGCTTAGAGCTTAATTGCTGAATCTGGTGCTGGGATCCAACATGTTTTAAATATG | 80 bp dsDNA substrate |
| PSOL2243 | CATATTTAAAACATGTTGGATCCCAGCACCAGATTCAGCAATTAAGCTCTAAGCCATGAATTCAAATGACCTCTTATCAA | 80 bp dsDNA substrate |
| PSOL4642 | GATCCTCTAGTACTTCTC | PCR primer for 2 kb dsDNA substrate |
| PSOL6134 | TCTACCTCAAGACTGGTCAG | PCR primer for 2 kb dsDNA substrate |
| PSOL7112 | ATCGTagatctATGGCAGCAAACTCTTCAGGA | PCR primer for 315 bp dsDNA substrate |
| PSOL7113 | TGCATggtaccTCATTCTGGGTCAAGGCGAG | PCR primer for 315 bp dsDNA substrate |
| PSOL7287 | GACGTCATAGACGATTACATTGCTAGGACATGCTGTCTAGAGACTATCGC | Y shaped substrate with 19-base flaps |
| PSOL7288 | GCGATAGTCTCTAGACAGCATGTCCTAGCAAGCCAGAATTCGGCAGGCTA | Y shaped substrate with 19-base flaps |
| PSOL7341 | AGGAGGAAAGGACGTCATAGACGATTACATTGCTAGGACATGCTGTCTAGAGACTATCGC | Y shaped substrate with 29-base flaps |
| PSOL7342 | GCGATAGTCTCTAGACAGCATGTCCTAGCAAGCCAGAATTCGGCAGGCTAGAAAGGAGGA | Y shaped substrate with 29-base flaps |
| PSOL7345 | AGAGGAAAGGAGAAAGGAGGAAAGGAAGAAAGGAGGAAAGGACGTCATAGACGATTACATTGCTAGGACATGCTGTCTAGAGACTATCGC | Y shaped substrate with 44-base flaps |
| PSOL7346 | GCGATAGTCTCTAGACAGCATGTCCTAGCAAGCCAGAATTCGGCAGGCTAGAAAGGAGGAAAGAAGGAAAGGAGGAAAGAGGAAAGGAGA | Y shaped substrate with 44-base flaps |
| PSOL7369 | GCGATAGTCTCTAGACAGCATGTCCTAGCAA | 19-base 5’ overhang substrate |
| PSOL7370 | TTGCTAGGACATGCTGTCTAGAGACTATCGC | 19-base 3’ overhang substrate |
| PSOL7288 | GCGATAGTCTCTAGACAGCATGTCCTAGCAAGCCAGAATTCGGCAGGCTA | 19-base 3’ overhang substrate |
| PSOL7343 | AGAGGAAAGGAAGAAAGGAGGAAAGGACGTCATAGACGATTACATTGCTAGGACATGCTGTCTAGAGACTATCGC | 44-base 5’ overhang substrate |
| PSOL7370 | TTGCTAGGACATGCTGTCTAGAGACTATCGC | 44-base 3’ overhang substrate |
| PSOL7344 | GCGATAGTCTCTAGACAGCATGTCCTAGCAAGCCAGAATTCGGCAGGCTAGAAAGGAGGAAAGAAGGAAAGGAGA | 44-base 3’ overhang substrate |
